# Individuating multiple (not one) persons reduces implicit racial bias

**DOI:** 10.3389/fpsyg.2022.939811

**Published:** 2022-07-22

**Authors:** Miao Qian, Gail D. Heyman, Mingzhan Wu, Genyue Fu

**Affiliations:** ^1^Department of Psychology, University of Detroit Mercy, Detroit, MI, United States; ^2^Department of Psychology, University of California, San Diego, San Diego, CA, United States; ^3^Department of Psychology, Hangzhou Normal University, Hangzhou, China

**Keywords:** implicit bias, individuation, differentiation, racial bias, explicit bias

## Abstract

Individuation training that helps humans see multiple other-race targets as distinct rather than as interchangeable can reduce children’s implicit racial bias in the form of more negative other-race associations than own-race associations. However, little is known about which aspects of these interventions are critical for their effectiveness. The present research examines whether children need to learn to differentiate among *multiple* other-race individuals for these interventions to reduce their level of implicit racial bias, or whether differentiating a *single* other-race individual is sufficient. We addressed this question among 4-to-6-year-old Chinese children (*N* = 66, 31 girls) who engaged in coordinated movement with Black instructors for 2 min. There were two between-subject conditions: in a *differentiation* condition, there were four different Black instructors, and children had to learn to tell them apart, and in a *no-differentiation* condition, there was only one Black instructor. Implicit bias was measured using the IRBT, an implicit association test that was developed based on the IAT but is appropriate for young children. We found a reduction in implicit bias against Black people after this interaction in the differentiation condition, but not in the no-differentiation condition. These findings suggest that learning to differentiate among multiple other-race individuals plays a critical role in reducing children’s implicit racial bias.

## Introduction

Racial biases exert far-reaching negative impacts on individuals and institutions (Green et al., 2007; Pascoe and Smart Richman, 2009; Benson and Fiarman, 2020; Chae et al., 2020; Chetty et al., 2020). In recent years, researchers have been developing a range of strategies to address this issue at multiple levels (Alhejji et al., 2016; Sue et al., 2019; Paluck et al., 2021), including some that are at the level of the individual and that target implicit bias. These efforts have met with limited success when targeted at adults (Lai et al., 2014, 2016; FitzGerald et al., 2019; Forscher et al., 2019), but recent research suggests that interventions may be more effective when initiated in childhood before attitudes become entrenched (Xiao et al., 2015; Gonzalez et al., 2017; Qian et al., 2019b, 2020). One successful approach has involved individuation training, in which children learn to identify multiple people of different races as distinct individuals rather than as interchangeable (Xiao et al., 2015; Qian et al., 2017, 2019b). However, there are important questions about the mechanisms that underlie this effect. In the present research, we examine the critical question of whether the effect depends on differentiation among *multiple* other-race individuals, which would suggest that individuation is necessary, or whether a *single* other-race individual is sufficient.

Implicit racial bias is one form of racial biases that refers to attitudes and beliefs that are more automatic and less consciously accessible (Greenwald and Banaji, 1995; Bargh, 1999). Existing research on implicit racial bias often relies on indirect methods, such as the Implicit Association Test (IAT; Greenwald et al., 1998), which examines the strength of association between a race concept (e.g., Black or White) and an attribute (e.g., good or bad) based on the relative speed when making different pairings between the concept and attribute.

Developmental scientists have been increasingly interested in the development of implicit racial bias in childhood and have developed new assessments that minimize cognitive demands (Baron and Banaji, 2006; Cvencek et al., 2011; Qian et al., 2016). For example, Baron and Banaji (2006) first developed a child version of the IAT (Child-IAT), which examines associations between graphics of own- versus other-race faces, and positive versus negative words that are read out loud. These child-friendly IATs generally indicate a pattern of implicit racial bias favoring racial ingroup among children as young as age 6 years (Baron and Banaji, 2006; Newheiser and Olson, 2012; for evidence of implicit bias in 3-year-old children, see Qian et al., 2016).

One strategy that may be effective for reducing implicit racial bias is giving people the opportunity to individuate other-race persons (Lebrecht et al., 2009; Xiao et al., 2015; Qian et al., 2017, 2019b). According to the Perceptual-Social Linkage hypothesis, individuation prevents children from lumping other-race persons together and applying negative stereotypes broadly to an other-race category as a whole because implicit racial bias stems from children’s early asymmetrical experience of being skilled at differentiating own-race faces and poor at differentiating other-race faces (Lee et al., 2017). The “sameness” not only occurs at the level of perceptual recognition but also conceptual level, termed the “outgroup homogeneity effect” (Judd and Park, 1988). That is, outgroup members tend to be viewed as having less variability in attitudes, values, and personality traits than ingroup members (Judd and Park, 1988; Guinote et al., 2007; Shilo et al., 2019). Perceiving outgroup members as homogenous is closely associated with stereotype endorsement (Ryan et al., 1996; Hewstone and Hamberger, 2000) and dehumanization (for a review, see Haslam, 2006). In line with these correlational findings, experimental evidence has documented that increasing perceived group variability can reduce stereotypes and prejudice. For example, in a series of four laboratory and field experiments, Brauer and Er-rafiy (2011) did manipulations that increased the perceived variability of a target group, which resulted in reduced prejudice and discrimination toward the outgroup.

There is some empirical evidence supporting the Perceptual-Social Linkage hypothesis. Xiao et al. (2015) first documented the effectiveness of individuation training, in which young children learned to distinguish among multiple other-race faces. In their study, 4- to 6-year-old Chinese children learned to tell five Black adult faces apart from each other. These researchers found that this training led to a reduction in implicit anti-Black bias. Qian et al. (2017) replicated these findings and further documented that merely exposing children to multiple other-race faces without requiring children to learn to tell them apart from each other was not effective.

Further support for the Perceptual-Social Linkage hypothesis comes from findings showing that coordinated movement involving differentiating multiple other-race members reduces children’s racial bias. This was documented using an individuated synchronous movement paradigm, in which children learned the names of four other-race instructors and performed physical exercises such as clapping hands with them.^1^ Five-year-old Chinese children who were given this intervention with Black instructors showed a decrease in implicit bias. Their counterparts in a control condition with Chinese instructors showed no such decrease in implicit bias.

Although findings from Qian et al. (under review; See Footnote 1) showed that the coordinated movement approach can work, they do not answer the question of whether differentiating among multiple other-race individuals is critical to successfully reducing bias. It may not be, given that providing identifying information about a single outgroup member can motivate people to move beyond the automatic, category-based process when judging others (Fiske and Neuberg, 1990). Some evidence suggests that providing identifying information about a single outgroup member may be beneficial to reducing racial bias in childhood: Rhodes et al. (2012) found that descriptions about an individual group member (“*this Zarpie is scared of ladybugs*”) reduced 4-year-old children’s essentialist beliefs about the group as a whole. More direct support for this possibility comes from the evidence regarding the responsiveness of implicit bias to individuating information. According to the Associative-Propositional Evaluations (APE) model (Gawronski and Bodenhausen, 2006, 2011), implicit bias is sensitive to propositional information defined as a statement about the world that has an objective truth value (e.g., “The apple is red”). According to this definition, many types of individuating information about a person are propositional (e.g., “John is a fire chief”), and thus may influence implicit bias toward the person. This influence is likely to be especially strong when the diagnosticity of the individuating information increases (Cone and Ferguson, 2015; Rubinstein et al., 2018). Thus, it is plausible that learning the name and the specific activities that one other-race person engages in could be sufficient to reduce implicit racial bias.

The present study adapted the individuated synchronous movement paradigm used by Qian et al. (under review; Footnote 1) to test whether providing individuating information about one single outgroup member would be sufficient to reduce children’s implicit racial bias toward the racial group of that individual or whether distinguishing between multiple individuals was necessary. We did this by comparing two groups: One group was led by four other-race instructors and the other group was identical, except there was only one other-race instructor.

Based on prior research examining interventions designed to reduce anti-Black bias among Chinese children (Xiao et al., 2015; Qian et al., 2017, 2019b), we predicted that differentiating multiple outgroup members would be a critical component in reducing implicit racial bias. We also assessed children’s explicit racial bias to explore whether the effects would be similar or different than for implicit bias.

## Materials and methods

### Participants

The sample included 66 Chinese children (35 boys, 31 girls; *M*_age_ = 5.90 years, *SD* = 0.48) who participated in the study. Participants were Han Chinese recruited from a range of socioeconomic backgrounds (parental education levels ranged from Grade 9 to postgraduate level, with the median education level being Grade 12). This research was approved by the [Blinded for review] research ethics committee.

We did *a priori* power analysis by G*Power (Faul et al., 2009), and it indicated that 31 subjects in each group were required to have 95% power for detecting a medium-sized effect (average *f* = 0.25, *f* ranges from 0.18 to 0.41 based on previous research with a similar design; Qian et al., 2017, 2019a, 2020), when employing the traditional 0.05 criterion of statistical significance.

Participants were randomly assigned to a *differentiation* condition (*N* = 33, 15 girls), in which they were asked to play a 2-min coordinated movement game with four Black instructors, and/or a *no-differentiation* condition (*N* = 33, 16 girls), in which they were asked to play the same game with only one Black instructor for the same amount of time. We did not include an additional control condition with same race individuals in light of prior research documenting no effect of such interactions on implicit bias (Qian et al., 2017, 2019b; Yu et al., 2021).

### Procedure

Participants were assessed individually in a quiet room at their school. One male Chinese graduate student tested all participants in Mandarin. In the introductory phase of the study, this experimenter taught children a coordinated movement game, in which they were asked to imitate four simple body movements: clapping hands, head movements, jumping jacks, and stepping. After children learned how to play the game, they engaged in a condition manipulation that took place *via* video and lasted a total of 2 min. The nature of the manipulation depended on the condition to which they were assigned.

In the differentiation condition, children were introduced to four separate videos one by one. Each video lasted for 30 s, depicting a different Black male who performed one of the four simple body movements. Before watching each video, children were first introduced to the person’s name (“*This is Dave. You are going to play the movement game with him.*”). They were asked to repeat the person’s name. Upon remembering the Black person’s name, children then engage in coordinated movement with the person by imitating the actions they had learned in the introductory phase. After each video, children were asked to repeat the person’s name and were reminded of the name if they were not able to recall it. After stating each person’s name, they were tested on the names of all previous instructors they had interacted with. For example, on the third trial, they interacted with an instructor named Adam. Participants repeated his name, stepped together with him, and then were asked to recall his name (“What was the name of the person who stepped with you?”). They were then asked to recall the names of the instructors in the previous two videos. Specifically, they were asked, “What was the name of the person who clapped hands with you?” (shown in the first video), and “What was the name of the person who moved heads with you?” (shown in the second video). Children were corrected on any names they got wrong.

In the no-differentiation condition, children engaged in the same activities, but there was only one Black male instructor: children in this condition only had to learn the name of that one individual and do the coordinated movements with him. As in the differentiation condition, participants were asked to say the name of the person before and after each video. Across both conditions, implicit and explicit racial biases toward Black people were assessed before and after the condition manipulation.

### Measures

#### Implicit racial bias measurement

Implicit bias was measured *via* the Implicit Racial Bias Test (IRBT, Qian et al., 2016), a preschooler-friendly variant of the Implicit Association Test (IAT, Greenwald et al., 1998). Following the same logic as the IAT, the IRBT assesses differences in reaction time to map own- and other-race faces with positive and negative attributes, such as a smile or a frown. These adaptions are consistent with previous child-friendly IAT variants to reduce cognitive demands (Baron and Banaji, 2006; Cvencek et al., 2011) and have shown comparable reliability and predictive validity as what was previously seen with adults (Williams and Steele, 2016; Rae and Olson, 2018). The IRBT has been validated in children in several different cultures (Qian et al., 2016; Setoh et al., 2019; Singh et al., 2020). We used Cronbach’s alpha score to estimate the internal consistency of the IRBT, and found that the task had a reliability with *α* = 0.86 (for congruent pairings, *α* = 0.79, and for incongruent pairings, *α* = 0.74), indicating acceptable internal consistency.

The IRBT was conducted on a Microsoft Surface Pro tablet with a 12-inch touch screen, using E-prime 2.0 (Psychology Software Tools, Sharpsburg, PA). Children were instructed to tap either a line drawing of a smile or a line drawing of a frown. In the “congruent” block, children were instructed to tap the smile when they saw a Chinese face and frown when they saw a Black face. In the “incongruent” block, they were instructed to do the opposite: tapping a smile when they saw a Black face and a frown when they saw a Chinese face. After each trial, children were given feedback and were required to correct their response each time they made an incorrect response.

There were 8 practice trials to allow for familiarization with the procedure and 20 test trials for each congruent and incongruent block. Children were instructed to complete each trial as fast as they could. Half of the participants started with the “congruent” block, and the other half started with the “incongruent” block.

Color photos of 20 Chinese faces (10 females) and 20 Black faces (10 females) were chosen from a face database (Ge et al., 2009). Ten Chinese faces and 10 Black faces were randomly chosen and presented in the congruent block. The other 10 Chinese faces and 10 Black faces were randomly presented in the incongruent block. The same set of photos was used for the IRBT at posttest. All photos were cropped into an elliptical shape (hair removed for consistency). These photos were also standardized at 480 pixels (17 cm) wide and 600 pixels (21 cm) tall, with 72 pixels per inch resolution.

#### Explicit racial bias measurement

Explicit bias was measured by a forced-choice task (see similar tasks used in Baron and Banaji, 2006; Dunham et al., 2006; Qian et al., 2016). In the task, children were asked to self-report their racial preference for interacting with either an own-race or an other-race individual in daily scenarios. Four scenarios were presented in a random order for each child: interacting with a doctor, music teacher, swimming coach, and tour guide (e.g., *This summer your mother will take you to a swimming class. In the class, you can choose one person to coach you to swim. Which one would you like to choose?*). Each scenario was presented along with depictive line drawings. Chinese and Black individuals were represented using cartoon faces. The left-right positions of the Chinese and Black faces were counterbalanced across participants and scenarios.

## Results

### Implicit racial bias

Consistent with previous IAT research (Greenwald et al., 2003), we calculated D scores to reflect children’s implicit racial bias toward Black people. The D score is the difference between the mean response latencies of contrasted conditions divided by the standard deviation across conditions (Greenwald et al., 2003; Qian et al., 2016). Consistent with previous IRBT studies (Qian et al., 2016, 2019a), participants with 10% responses faster than 300 ms, an error rate that exceeded 60%, or an average response latency 3 *SD* above the mean latency across the entire task were removed. These criteria led to 5 children being excluded and left 61 participants for analysis on the implicit task (*n* = 30 in the *differentiation* condition and *n* = 31 participants in the *no-differentiation* condition).

To examine whether Chinese children showed implicit bias against Black people, we performed a one-sample *t*-test to compare their implicit bias scores at pretest to zero (no bias). Replicating previous results with Chinese children (Qian et al., 2016, 2019a), we found that they displayed a significant implicit bias against Black people at pretest, *D* = 0.49, *SD* = 0.35, *t*(60) = 10.92, *p* < 0.001, Cohen’s *d* = 1.40, 95% *CI* [0.40, 0.58].

**Figure 1:**
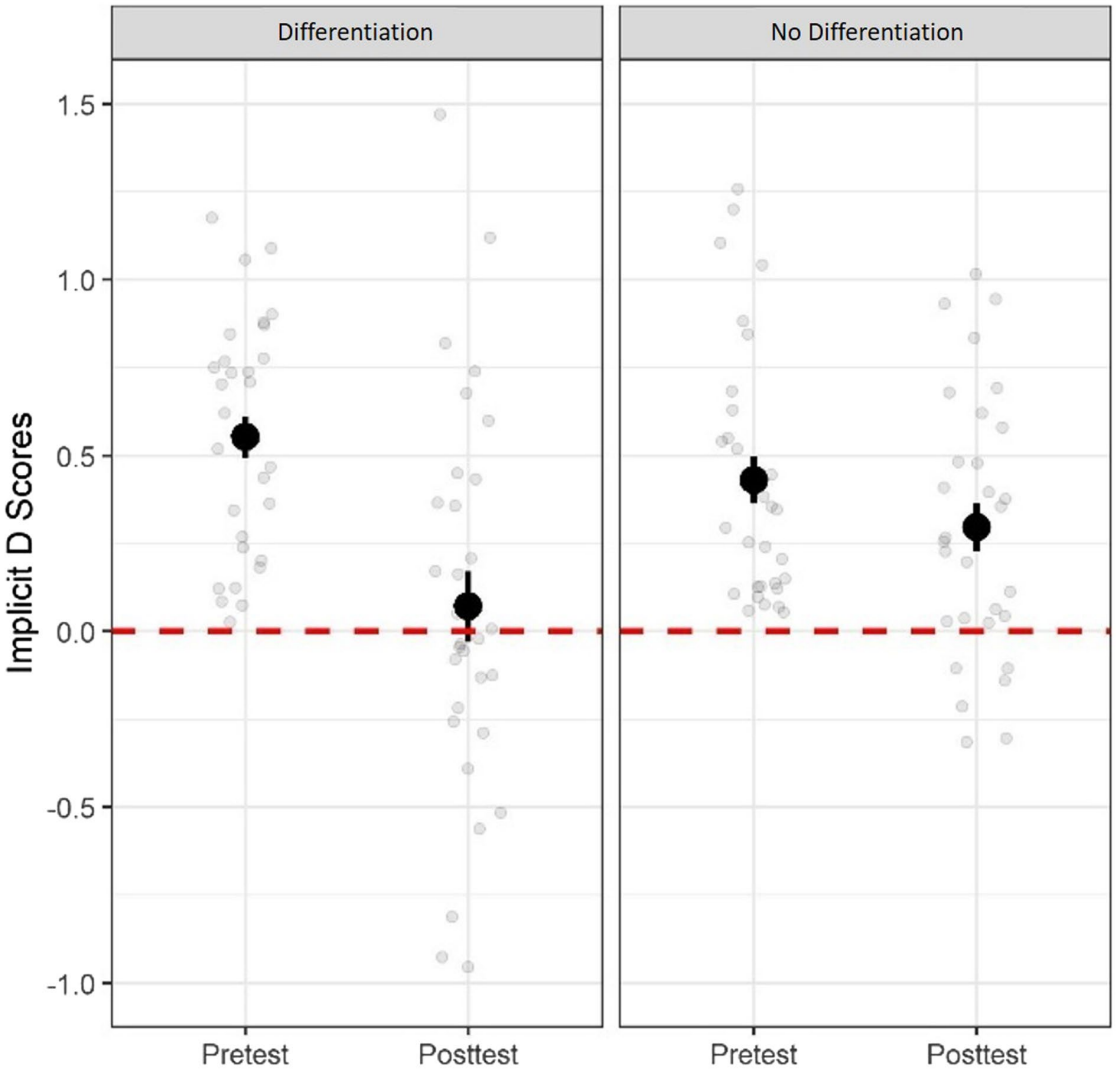
Implicit racial bias at pretest and posttest for the differentiation and no-differentiation conditions. Grey dots represent each participant’s score. Error bars represent standard errors. Red dashed lines represent no bias.

To answer the question of whether providing individuating information about one single outgroup member would be sufficient to reduce children’s implicit racial bias or whether distinguishing between multiple individuals was critical, a mixed factorial ANOVA was conducted. This was conducted with condition (differentiation, no-differentiation) as a between-subject variable and test time (pretest or posttest) as a repeated-measure variable on implicit racial biases. We found a significant Condition x Test Timing interaction, *F*(1, 59) = 5.21, *p* = 0.026, partial *η*^2^ = 0.08 (see Figure 1). *Post-hoc* paired sample *t*-tests showed that children in the differentiation condition showed a reduction in implicit anti-Black racial bias from pretest (*M* = 0.55, *SD* = 0.33) to posttest (*M* = 0.06, *SD* = 0.56), *t*(30) = 4.10, *p* < 0.001, Cohen’s *d* = 0.73, 95% *CI* [0.25, 0.73], unlike children in the no-differentiation condition, who showed no significantly change from pretest (*M* = 0.43, *SD* = 0.37) to posttest (*M* = 0.30, *SD* = 0.38), *t*(29) = 1.36, *p* = 0.185, Cohen’s *d* = 0.07, 95% *CI* [−0.07, 0.34]. These results suggest that performing coordinated movement with multiple Black people, but not with only one Black person, decrease participants’ implicit anti-Black bias.

To further explore whether children showed implicit racial bias after the differentiation and no-differentiation manipulations, we conducted one-sample *t-*tests to compare their implicit D scores to zero. We found that implicit D scores was significantly above zero for children in the no-differentiation condition, *D* = 0.30, *SD* = 0.38, *t*(29) = 4.29, *p* < 0.001, Cohen’s *d* = 0.38, 95% *CI* [0.15, 0.44], but not for children in the differentiation condition, *D* = 0.06, *SD* = 0.56, *t*(30) = 10.92, *p* = 0.538, Cohen’s *d* = 0.56, 95% *CI* [−0.14, 0.27]. These results suggest that performing coordinated movement with multiple Black people eliminated implicit anti-Black bias.

### Explicit racial bias

Choice of the Chinese individuals (own-race) over the Black individuals (other-race) was coded as 1, and choice of the Black individuals (other-race) over the Chinese individuals (own-race) was coded as 0 for each of the four scenarios. The scores were added up and divided by four to derive a proportion score, with 0.50 as the no-bias score.

To examine whether Chinese children showed explicit racial bias against Black people, we performed one-sample *t*-tests to compare their explicit bias scores at pretest to 0.50 (no bias). Consistent with Qian et al. (2016), we found that Chinese preschool-age children expressed a significant explicit bias against Black people at pretest, *M* = 0.78, *t*(60) = 32.14, *p* < 0.001, Cohen’s *d* = 1.38.

**Figure 2:**
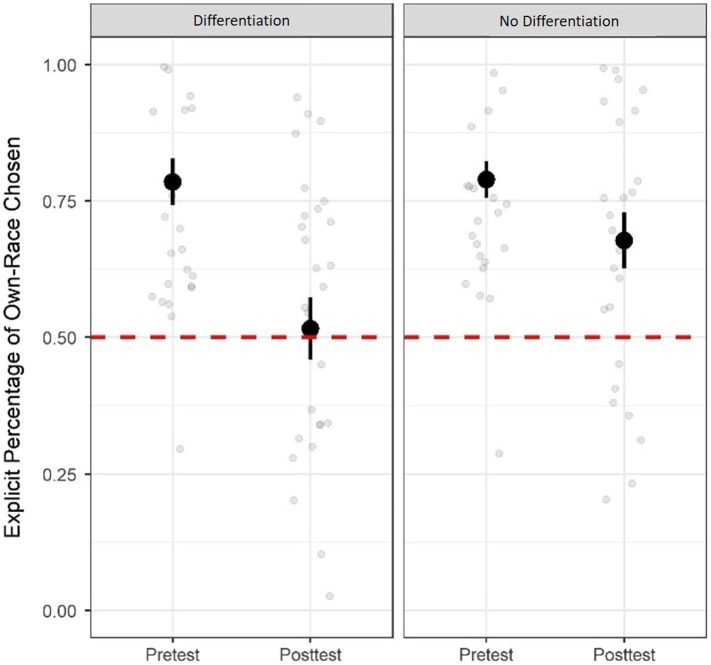
Explicit anti-Black bias at pretest and posttest for the differentiation and no-differentiation conditions. Grey dots represent each participant’s score. Error bars represent standard errors. Red dashed lines represent no bias.

To determine the effects of each condition on explicit anti-Black bias, we conducted the same mixed factorial ANOVA with condition (differentiation, no-differentiation) as a between-subject variable and test time (pretest or posttest) as a repeated-measure variable on explicit racial biases. We found a main effect of test timing on explicit racial bias, *F*(1, 59) = 19.00, *p* < 0.001, partial *η*^2^ = 0.24, suggesting that both conditions led to reductions in explicit racial biases from pretest (*M* = 0.79, *SD* = 0.21) to posttest (*M* = 0.60, *SD* = 0.27; see Figure 2). We also found a marginally significant Condition x Test Timing interaction, *F*(1, 59) = 3.27, *p* = 0.075, partial *η*^2^ = 0.05. *Post-hoc* paired sample *t*-test showed that children in the differentiation condition displayed less explicit bias than those in the no-differentiation condition at posttest, *t*(59) = 2.09, *p* = 0.041. These results suggest that both conditions were effective at decreasing children’s explicit anti-Black bias, but that the differentiation condition led to a greater reduction than the no-differentiation condition.

To further explore whether children showed explicit racial bias after the differentiation and no-differentiation manipulations, we conducted one-sample *t-*tests to compare their percentage of own-race chosen to 0.50 (no bias). We found that explicit racial bias was significantly above 0.50 for children in the no-differentiation condition, *M* = 0.68, *SD* = 0.28, *t*(29) = 3.44, *p* = 0.002, Cohen’s *d* = 0.32, 95% *CI* [0.07, 0.28], but not for children in the differentiation condition, *M* = 0.52, *SD* = 0.32, *t*(30) = 0.28, *p* = 0.781, Cohen’s *d* = 0.28, 95% *CI* [−0.10, 0.13]. These results suggest that performing coordinated movement with multiple Black people, in contrast to doing so with just one Black person, eliminated children’s explicit anti-Black bias.

## Discussion

Individuation training, in which children learn to distinguish the identities of multiple other-race individuals, has shown substantial promise in combating implicit racial bias (Xiao et al., 2015; Qian et al., 2017, 2019b). Although differentiating among multiple other-race individuals is often assumed to be a critical component of the success of this intervention, this has not yet been tested: it could be that it is sufficient to obtain the same results by learning the identifying information about a single other-race individual. We assessed this question in a sample of 4- to 6-year-old Chinese children. In a differentiation condition, participants performed physical exercises with four Black instructors and learned their names, and in a no-differentiation condition, they did the same thing with one Black instructor and learned his name.

As hypothesized, we found that only children in the differentiation condition, who interacted with multiple other-race individuals, showed a reduction in anti-Black implicit bias. This suggests that differentiation among multiple outgroup members is necessary to reduce implicit racial bias. These results are in line with prior work indicating that individuation training requiring children to learn to recognize multiple other-race faces reduces implicit bias (Xiao et al., 2015; Qian et al., 2017, 2019b; Qian et al., under review; See Footnote 1).

Although the focus of our research was on implicit bias, we also examined effects on explicit bias. We found that, as was the case with implicit bias, the differentiation condition was better than the no-differentiation condition at reducing explicit bias. This may be because interacting with multiple individuals of another race facilitates memory retrieval of multiple (instead of just one) exemplars when making judgments about the group (Dasgupta and Greenwald, 2001; Gawronski and Bodenhausen, 2006).

Even though we found that the differentiation condition was better at reducing explicit bias than the no-differentiation condition, the no-differentiation condition was still effective at reducing this form of bias. This suggests that providing identifying information about a single outgroup member without the experience of differentiation is sufficient to reduce explicit racial bias, a finding which is consistent with prior work with both adults (Harris and Fiske, 2006, 2007; Haslam and Bain, 2007) and children (Rhodes et al., 2012). One reason for this is that individuating information about a target person can lead the person to be perceived as more human (Harris and Fiske, 2006, 2007). For example, in one study, Haslam and Bain (2007) found that when adult participants received individuating information about a hypothetical student (i.e., initials, gender, and age), this led them to perceive the student as having more traits associated with human nature, such as the tendency to be helpful or nervous.

Another possible explanation is that coordinated movement with other-race members leads to a sense of interpersonal synchrony, which can increase feelings of interpersonal closeness and intergroup affiliation in infants and young children (Cirelli et al., 2014; Rabinowitch and Meltzoff, 2017; Qian et al., 2020). This possibility is in line with findings by Qian et al. (2020), who trained 4- to 6-year-old children to perform a clap and tap game to the same beat as an other-race person and found that this reduced children’s explicit racial bias toward the other-race group.

Our findings build on prior evidence indicating that the same intervention can have different effects on implicit and explicit racial bias (Qian et al., 2017, 2020; Yu et al., 2021) and raises important questions about why providing identifying information about a single outgroup member is sufficient to reduce explicit bias but not implicit racial bias. One possible reason is that implicit measures use exemplars of the race category (e.g., individual Black faces) as stimuli, while explicit measures assess responses to race categories (e.g., Black people in general), and it may be easier for children to generalize positive attitudes from an individual to the group than from one individual to another.

## Limitations and future directions

Although the present study provides evidence supporting the importance of differentiation in reducing implicit racial bias, many questions remain. Additional studies will be needed to better understand why differentiation is important. One possibility is that individuating information about a single person only affects implicit bias toward that person but does not impact attitudes toward the outgroup as a whole. Future research could potentially assess this possibility by including measures of implicit bias toward individual instructors.

Our research found beneficial effects of an intervention in which the contact was indirect (i.e., *via* video). Future research will be needed to determine whether the effects would be different if the contact was direct (i.e., in person rather than *via* video). One possibility is that direct contact eliminates any potential benefits of contact because such contact may lead to anxiety (Shelton et al., 2009; Trawalter et al., 2009). However, this has not been tested in contexts involving differentiation of other-race individuals.

Another future direction to explore is whether this intervention would achieve similar results when implemented in older children. Older children might need a larger intervention dosage, given that attitudes might be more entrenched with increased socialization experience (Waxman, 2021). However, it is also possible that the intervention might be more effective in older children, given evidence that some other types of interventions, such as exposure to counter-stereotypical exemplars, are less effective with younger children (Gonzalez et al., 2017).

Although our intervention is a promising approach to reduce implicit racial bias at the individual level, other approaches that target implicit bias at the structural or institutional level are also needed. Such approaches should also affect biases at the individual level given that there are cultural and structural influences on implicit bias, such as systematically biased social structures (Payne et al., 2017; Lai and Banaji, 2019).

In summary, the present study replicates prior research demonstrating the effectiveness of an individuated synchronous movement intervention for reducing both implicit and explicit racial bias. We also demonstrate that differentiating among multiple other-race individuals is a critical component to the success of this intervention in reducing children’s implicit racial bias. These findings pave the way for developing anti-bias interventions that are easy to implement in classrooms around the world.

## Data availability statement

Study materials, deidentified raw data and cleaned data, and data analysis scripts have been made publicly available via the Open Science Framework: https://osf.io/jksgf/.

## Ethics statement

The studies involving human participants were reviewed and approved by Hangzhou Normal University Institutional Review Board. Written informed consent to participate in this study was provided by the participants’ legal guardian/next of kin.

## Author contributions

MQ and GF conceived and planned the study. MW tested all participants. MQ and MW performed data analysis. MQ and GH wrote the manuscript. All authors contributed to the article and approved the submitted version.

## Funding

This research was supported by a National Science Foundation of China (31771227) grant to GF.

## Conflict of interest

The authors declare that the research was conducted in the absence of any commercial or financial relationships that could be construed as a potential conflict of interest.

## Publisher’s note

All claims expressed in this article are solely those of the authors and do not necessarily represent those of their affiliated organizations, or those of the publisher, the editors and the reviewers. Any product that may be evaluated in this article, or claim that may be made by its manufacturer, is not guaranteed or endorsed by the publisher.

## Footnotes

^1^Qian, M., Heyman, G. D., Compton, B. J., Yu, C., Lee, K., and Fu, G. (under review). Lasting Reduction in Children’s Implicit and Explicit Racial Bias Following Individuated Synchronous Movement with Other-Race People.
